# Alirocumab therapy in individuals with type 2 diabetes mellitus and atherosclerotic cardiovascular disease: analysis of the ODYSSEY DM-DYSLIPIDEMIA and DM-INSULIN studies

**DOI:** 10.1186/s12933-019-0951-9

**Published:** 2019-11-09

**Authors:** Kausik K. Ray, Stefano Del Prato, Dirk Müller-Wieland, Bertrand Cariou, Helen M. Colhoun, Francisco J. Tinahones, Catherine Domenger, Alexia Letierce, Jonas Mandel, Rita Samuel, Maja Bujas-Bobanovic, Lawrence A. Leiter

**Affiliations:** 1 Imperial Centre for Cardiovascular Disease Prevention, Department of Primary Care and Public Health, Imperial College, Kensington, London, SW7 2AZ UK; 2 Department of Clinical and Experimental Medicine, University of Pisa, Pisa, Italy; 3 Department of Internal Medicine I, University Hospital Aachen, Aachen, Germany; 4 l’institut du thorax, Department of Endocrinology, CHU Nantes, INSERM, 1413 Nantes, France; 5 University of Edinburgh, Edinburgh, Scotland, UK; 6 Department of Clinical Endocrinology and Nutrition (IBIMA), Hospital Virgen de la Victoria, University of Málaga, CIBER Fisiopatología de la Obesidad y Nutrición (CIBERobn), Instituto de Salud Carlos III, Málaga, Spain; 7 Sanofi, Gentilly, France; 8 Biostatistics and Programming Department, Sanofi, Chilly-Mazarin, France; 9 IviData Stats, Levallois-Perret, France; 10 Regeneron Pharmaceuticals, Inc., Tarrytown, New York, NY USA; 11 Sanofi, Paris, France; 12 Li Ka Shing Knowledge Institute, St. Michael’s Hospital, University of Toronto, Toronto, ON Canada

**Keywords:** Alirocumab, Atherosclerotic cardiovascular disease, Type 2 diabetes mellitus, Low-density lipoprotein cholesterol, Dyslipidemia

## Abstract

**Background:**

Individuals with diabetes often have high levels of atherogenic lipoproteins and cholesterol reflected by elevated low-density lipoprotein cholesterol (LDL-C), non-high-density lipoprotein cholesterol (non-HDL-C), apolipoprotein B (ApoB), and LDL particle number (LDL-PN). The presence of atherosclerotic cardiovascular disease (ASCVD) increases the risk of future cardiovascular events. We evaluated the efficacy and safety of the proprotein convertase subtilisin/kexin type 9 (PCSK9) inhibitor, alirocumab, among individuals with type 2 diabetes (T2DM), high LDL-C or non-HDL-C, and established ASCVD receiving maximally tolerated statin in ODYSSEY DM-DYSLIPIDEMIA (NCT02642159) and DM-INSULIN (NCT02585778).

**Methods:**

In DM-DYSLIPIDEMIA, individuals with T2DM and mixed dyslipidemia (non-HDL-C ≥ 100 mg/dL; n = 413) were randomized to open-label alirocumab 75 mg every 2 weeks (Q2W) or usual care (UC) for 24 weeks, with UC options selected before stratified randomization. In DM-INSULIN, insulin-treated individuals with T2DM (LDL-C ≥ 70 mg/dL; n = 441) were randomized in a double-blind fashion to alirocumab 75 mg Q2W or placebo for 24 weeks. Study participants also had a glycated hemoglobin < 9% (DM-DYSLIPIDEMIA) or < 10% (DM-INSULIN). Alirocumab dose was increased to 150 mg Q2W at week 12 if week 8 LDL-C was ≥ 70 mg/dL (DM-INSULIN) or non-HDL-C was ≥ 100 mg/dL (DM-DYSLIPIDEMIA). Lipid reductions and safety were assessed in patients with ASCVD from these studies.

**Results:**

This analysis included 142 DM-DYSLIPIDEMIA and 177 DM-INSULIN participants with ASCVD, including 95.1% and 86.4% with coronary heart disease, and 32.4% and 49.7% with microvascular diabetes complications, respectively. At week 24, alirocumab significantly reduced LDL-C, non-HDL-C, ApoB, and LDL-PN from baseline versus control. This translated into a greater proportion of individuals achieving non-HDL-C < 100 mg/dL (64.6% alirocumab/23.8% UC [DM-DYSLIPIDEMIA]; 65.4% alirocumab/14.9% placebo [DM-INSULIN]) and ApoB < 80 mg/dL (75.1% alirocumab/35.4% UC and 76.8% alirocumab/24.8% placebo, respectively) versus control at week 24 (all *P *< 0.0001). In pooling these studies, 66.4% (alirocumab) and 67.0% (control) of individuals reported treatment-emergent adverse events. The adverse event pattern was similar with alirocumab versus controls.

**Conclusions:**

Among individuals with T2DM and ASCVD who had high non-HDL-C/LDL-C levels despite maximally tolerated statin, alirocumab significantly reduced atherogenic cholesterol and LDL-PN versus control. Alirocumab was generally well tolerated.

*Trial registration* Clinicaltrials.gov. NCT02642159. Registered 30 December 2015 and Clinicaltrials.gov. NCT02585778. Registered 23 October 2015

## Background

The leading cause of morbidity and mortality for individuals with diabetes is atherosclerotic cardiovascular disease (ASCVD). ASCVD may present as acute thrombotic manifestations of vascular disease, such as acute coronary syndrome (ACS), myocardial infarction (MI), unstable angina, stroke, and transient ischemic attack, or as occlusive manifestations of atherosclerotic vascular disease, such as stable angina and claudication [1, 2]. Atherogenic dyslipidemia is often observed in individuals with type 2 diabetes mellitus (T2DM), contributing to a higher risk of ASCVD [3]. This atherogenic dyslipidemia consists of an excess of atherogenic apolipoprotein B (ApoB)-containing lipoproteins, which is reflected by higher circulating ApoB, non-high-density lipoprotein cholesterol (non-HDL-C), and low-density lipoprotein particle number (LDL-PN) [1, 3, 4]. In addition, triglycerides are elevated and levels of HDL-C are low [3]. Therefore, lowering atherogenic cholesterol cargo is a major aim in many guidelines.

The 2019 American Diabetes Association (ADA) and the 2019 European Society of Cardiology (ESC)/European Atherosclerosis Society (EAS) guidelines recommend additional low-density lipoprotein-lowering therapy, such as ezetimibe or a proprotein convertase subtilisin/kexin type 9 (PCSK9) inhibitor, for patients who have diabetes and ASCVD if low-density lipoprotein cholesterol (LDL-C) goals are not met on the maximally tolerated statin dose [5, 6]. In the 2018 US cholesterol guidelines from the American College of Cardiology, American Heart Association, 2019 ESC/EAS guidelines, and others, individuals with clinical ASCVD are categorized as being at very high risk for cardiovascular (CV) events if they have additional risk factors, including diabetes and elevated LDL-C. Very high risk individuals who are on maximally tolerated LDL-C-lowering therapy with an LDL-C level of ≥ 70 mg/dL (≥ 1.8 mmol/L), or have a non-HDL-C level of ≥ 100 mg/dL (≥ 2.6 mmol/L), may add a PCSK9 inhibitor following a discussion with their clinician about the overall benefit, safety, and cost [2, 6]. The ODYSSEY OUTCOMES trial investigated the role of the PCSK9 inhibitor, alirocumab, in improving CV outcomes after ACS in patients on high-intensity statin therapy [7]. Results demonstrated that the risk of recurrent ischemic CV events was lower among those who were treated with alirocumab than placebo in this patient population. Alirocumab has further been assessed in two dedicated randomized studies in individuals with diabetes and hypercholesterolemia on maximally tolerated statin and either established ASCVD or additional CV risk factors: ODYSSEY DM-DYSLIPIDEMIA and DM-INSULIN [4, 8]. In this analysis, we evaluated the efficacy and safety of alirocumab versus control among individuals with T2DM, established ASCVD, and elevated non-HDL-C or LDL-C despite receiving maximally tolerated statin in the DM-DYSLIPIDEMIA and DM-INSULIN studies.

## Methods

This analysis included individuals with established ASCVD receiving maximally tolerated statin who were enrolled in the DM-DYSLIPIDEMIA and DM-INSULIN studies. ASCVD was defined as coronary heart disease (CHD; acute and silent MI, and unstable angina), ischemic stroke, or peripheral artery disease (PAD).

The DM-DYSLIPIDEMIA (NCT02642159) is a Phase IIIb/IV randomized, open-label, parallel group, multicenter, multinational clinical trial [9]. Individuals (n = 413) aged ≥ 18 years with T2DM and mixed dyslipidemia whose non-HDL-C level was not adequately controlled (≥ 100 mg/dL [> 2.59 mmol/L]) despite stable maximally tolerated statin dose for ≥ 4 weeks prior to screening visit, without other lipid-lowering therapies (LLTs), and who had either a documented history of ASCVD and/or at least one additional CV risk factor were included. Mixed dyslipidemia was defined as non-HDL-C ≥ 100 mg/dL (≥ 2.59 mmol/L) and triglycerides ≥ 150 mg/dL (≥ 1.70 mmol/L) and < 500 mg/dL (< 5.65 mmol/L). Study participants were also required to have a glycated hemoglobin (HbA1c) level of < 9% (74.9 mmol/mol). Eligible individuals were randomized to open-label alirocumab 75 mg (with blinded dose increase to 150 mg at week 12 if week 8 non-HDL-C was ≥ 100 mg/dL [≥ 2.59 mmol/L]) or usual care (UC) every 2 weeks for 24 weeks, with UC options selected before stratified randomization based on the investigator’s preference for each participant. The following five UC options were included in the study: continued use of maximally tolerated statin therapy with no additional LLT, fenofibrate, ezetimibe, omega-3 fatty acid, and nicotinic acid, reflecting variability in regional practice and therapeutic options available at the time the study was conducted. Safety was assessed through adverse events, laboratory parameters and vital signs. Treatment-emergent adverse events were defined as any event that developed, worsened or became serious during the period from first to last open-label dose of alirocumab plus 70 days (if randomized to alirocumab) or, if randomized to UC, 70 days after the last UC treatment or study day 225 (whichever came first).

In the DM-INSULIN (NCT02585778) phase IIIb, randomized, double-blind, placebo controlled, parallel group, multicenter trial, insulin-treated individuals aged ≥ 18 years with T2DM (n = 441) or type 1 diabetes mellitus (T1DM; n = 76) diagnosed ≥ 1 year prior to screening, and who had either a documented history of ASCVD and/or at least one additional CV risk factor were randomized in a 2:1 double-blind fashion to alirocumab or placebo for 24 weeks [4]. Study participants were also required to have a HbA1c level < 10% (86 mmol/mol). Alirocumab-treated individuals received 75 mg every 2 weeks (Q2W), with blinded dose increase to 150 mg Q2W at week 12 if week 8 LDL-C was ≥ 70 mg/dL (≥ 1.81 mmol/L). Statins and other LLTs remained stable throughout the duration of the study. Primary safety endpoints were assessed up to week 32 through treatment-emergent adverse events (TEAE) reports, laboratory data, product complaints, and vital signs. Participants with T1DM were not included in the current analysis due to the low number of individuals with established ASCVD in this group (alirocumab: n = 11; placebo: n = 5).

Due to the substantial differences in the patient populations from DM-DYSLIPIDEMIA and DM-INSULIN, as well as the methodological differences between the two studies, efficacy was analyzed separately. The efficacy analysis included week 24 percentage reduction from baseline in non-HDL-C, calculated LDL-C, ApoB, triglyceride-rich lipoproteins (TGRL), and LDL-PN; the percentage of individuals achieving non-HDL-C < 100 mg/dL (< 2.59 mmol/L), LDL-C < 70 mg/dL (< 1.81 mmol/L), and ApoB < 80 mg/dL at week 24. TGRL was defined as non-HDL-C minus measured LDL-C if measured LDL-C not missing; non-HDL-C minus calculated LDL-C if measured LDL-C missing and calculated LDL-C not missing, using fasting samples first, or if fasting sample missing using non-fasting measurements. Efficacy data were analyzed with an intention-to-treat approach, including all randomized individuals with a non-HDL-C (DM-DYSLIPIDEMIA) or LDL-C (DM-INSULIN) value at baseline and at least one value post-baseline up to week 24. Safety data were pooled due to the small sample size, however separate adverse event outcomes are also reported.

### Statistical analysis

This was a post hoc analysis with similar statistical methods to those used in the primary DM-DYSLIPIDEMIA and DM-INSULIN studies [4, 8]. Percent changes from baseline in non-HDL-C, HDL-C, LDL-C, Apo-B and LDL-PN at week 24 were derived and compared between treatment groups using a mixed-effects model with repeated measures (MMRM), which accounts for missing data and utilizes every lipid values at week 0, 8, 12, 20 and 24. For TGRL, as normal distribution assumption wasn’t satisfied, their percent changes from baseline were estimated by robust regressions preceded by multiple imputations to handle missing data: combined estimates for means and standard errors (SE) are obtained by combining adjusted means and SE from robust regression model analyses of the different imputed datasets, using Rubin formulae.

The proportion of individuals achieving the different goals at week 24 were analyzed by multiple imputation followed by a logistic regression: the logistic regression included the treatment group and the UC stratum (for DM-DYSLIPIDEMIA) as main effects and the corresponding baseline value as covariate. Missing values were addressed using a multiple imputation approach and the logistic regressions were repeatedly performed in the datasets containing both observed and imputed lipid values and combined using Rubin formulae to allow for the treatment comparison. Analyses were in the intent-to-treat populations and for DM-DYSLIPIDEMIA, analyses are adjusted on the UC stratum. Descriptive analyses were performed for baseline, other efficacy, and safety analyses.

## Results

This analysis included 142 individuals from the DM-DYSLIPIDEMIA trial and 177 individuals from the DM-INSULIN trial, all of whom had established ASCVD and T2DM (Table 1). Overall, 93.7% of pooled individuals from DM-DYSLIPIDEMIA and 89.3% of individuals from DM-INSULIN had a history of hypertension, and 18.3% and 28.2% had chronic kidney disease on top of ASCVD, respectively. In total, 13.4% of pooled individuals from DM-DYSLIPIDEMIA and 20.3% of individuals from DM-INSULIN demonstrated a history of ischemic stroke; 7.0% and 10.7% had PAD, respectively; 95.1% and 86.4% had CHD, respectively; and 32.4% and 49.7% had microvascular diabetes complications, respectively.

**Table 1:** Baseline characteristics (randomized population)

	DM-DYSLIPIDEMIA		DM-INSULIN	
	Alirocumab (n = 95)	UC (n = 47)	Alirocumab (n = 119)	Placebo (n = 58)
Age, years, mean (SD)	64.9 (9.1)	65.4 (8.1)	66.2 (8.7)	64.9 (8.9)
Gender, male, n (%)	65 (68.4)	31 (66.0)	79 (66.4)	32 (55.2)
BMI, kg/m^2^, mean (SD)	33.0 (5.4)	32.7 (4.9)	32.6 (4.5)	33.4 (5.8)
CHD, n (%)	90 (94.7)	45 (95.7)	102 (85.7)	51 (87.9)
Acute MI	43 (45.3)	20 (42.6)	59 (49.6)	18 (31.0)
Silent MI	5 (5.3)	1 (2.1)	4 (3.4)	4 (6.9)
Unstable angina	15 (15.8)	9 (19.1)	15 (12.6)	4 (6.9)
Coronary revascularization	77 (81.1)	35 (74.5)	80 (67.2)	37 (63.8)
Other clinically significant CHD^a^	20 (21.1)	14 (29.8)	31 (26.1)	15 (25.9)
Ischemic stroke, n (%)	14 (14.7)	5 (10.6)	27 (22.7)	9 (15.5)
PAD, n (%)	6 (6.3)	4 (8.5)	13 (10.9)	6 (10.3)
HTN^b^, n (%)	89 (93.7)	44 (93.6)	105 (88.2)	53 (91.4)
CKD^c^, n (%)	15 (15.8)	11 (23.4)	37 (31.1)	13 (22.4)
Diabetes target organ damage^d^, n (%)	31 (32.6)	15 (31.9)	60 (50.4)	28 (48.3)
Statin, n (%)	80 (84.2)	41 (87.2)	92 (77.3)	42 (72.4)
Low intensity	6 (7.5)	0	3 (3.3)	1 (2.4)
Moderate intensity	21 (26.3)	20 (48.8)	46 (50.0)	24 (57.1)
High intensity	53 (66.3)	21 (51.2)	43 (46.7)	16 (38.1)
LLT other than statin^e^, n (%)	0	2 (4.3)	34 (28.6)	11 (19.0)
HbA1c, %, mean (SD)	7.0 (0.8)	7.2 (0.8)	7.5 (0.9)	7.4 (1.0)
FPG, mg/dL [mmol/L], mean (SD)	144.1 (39.3) [8.0 (2.2)]	152.6 (41.7) [8.5 (2.3)]	162.6 (52.5) [9.0 (2.9)]	146.7 (45.2) [8.1 (2.5)]
Insulin, n (%)	40 (42.1)	19 (40.4)	119 (100)	57 (98.3)^f^
Non-insulin GLT, n (%)				
Biguanides	72 (75.8)	34 (72.3)	57 (47.9)	33 (56.9)
Sulfonylureas	29 (30.5)	18 (38.3)	11 (9.2)	7 (12.1)
DPP-4 inhibitor	12 (12.6)	8 (17.0)	21 (17.6)	7 (12.1)
GLP-1 receptor agonist	16 (16.8)	6 (12.8)	11 (9.2)	8 (13.8)
SGLT2 inhibitor	10 (10.5)	5 (10.6)	10 (8.4)	11 (19.0)
Lipids, mg/dL [mmol/L], mean (SD)				
Non-HDL-C	156.5 (48.4) [4.05 (1.26)]	156.8 (43.3) [4.06 (1.12)]	142.8 (41.5) [3.70 (1.08)]	147.0 (54.9) [3.81 (1.42)]
LDL-C	108.3 (46.3) [2.81 (1.20)]	109.4 (44.0) [2.83 (1.14)]	107.2 (35.1) [2.78 (0.91)]	111.9 (46.4) [2.90 (1.20)]
ApoB	103.0 (26.7)	104.3 (27.8)	96.4 (25.1)	98.7 (32.0)
LDL-PN, nmol/L, mean (SD)	1400.2 (489.8)	1437.4 (479.4)	1339.5 (408.5)	1425.0 (467.9)

*Apo* apolipoprotein, *BMI* body mass index, *CHD* coronary heart disease, *CKD* chronic kidney disease, *DPP-4* dipeptidyl peptidase-4, *GLP-1* glucagon-like peptide-1, *GLT* glucose-lowering treatment, *FPG* fasting plasma glucose, *HbA1c* glycated hemoglobin, *HDL-C* high-density lipoprotein cholesterol, *HTN* hypertension, *LDL-C* low-density lipoprotein cholesterol, *LDL-PN* low-density lipoprotein particle number, *LLT* lipid-lowering therapy, *MI* myocardial infarction, *PAD* peripheral artery disease, *SD* standard deviation, *SGLT2* sodium glucose cotransporter 2

^a^Diagnosis by invasive/non-invasive testing

^b^Includes patients with established HTN on anti-HTN medication

^c^Defined as estimated glomerular filtration rate 15–60 mL/min/1.73 m^2^

^d^Defined as microalbuminuria, macroalbuminuria, retinopathy, and/or CKD

^e^LLT other than statins were not allowed per protocol in DM DYSLIPIDEMIA

^f^One individual in the placebo group was not receiving insulin at the time of randomization and remained without insulin treatment for the duration of the study

At baseline, pooled mean (standard deviation [SD]) non-HDL-C levels were 156.6 (46.6) mg/dL (4.06 [1.21] mmol/L) in DM-DYSLIPIDEMIA and 144.2 (46.2) mg/dL [3.73 (1.20) mmol/L] in DM-INSULIN; pooled LDL-C levels were 108.7 (45.4) mg/dL [2.81 (1.18) mmol/L] and 108.7 (39.1) mg/dL [2.82 (1.01) mmol/L], respectively; and pooled ApoB levels were 103.4 (26.9) mg/dL [2.67 (0.7) mmol/L] and 97.2 (27.6) mg/dL [2.51 (0.7) mmol/L]. The majority of individuals in both trials had diabetes for > 10 years, with a mean (SD) of 13.7 (8.8) years in alirocumab-treated individuals and 13.0 (9.7) years for UC individuals in DM DYSLIPIDEMIA. In DM-INSULIN, the mean (SD) duration of diabetes was 17.4 (8.3) years for alirocumab and 18.3 (9.2) years for placebo. Mean baseline HbA1c levels were 7.0% and 7.2% for alirocumab and placebo, respectively in DM-DYSLIPIDEMIA, and 7.5% for both alirocumab and placebo in DM-INSULIN. In DM-DYSLIPIDEMIA, 41.5% of pooled individuals were treated with insulin while 99.4% of individuals from DM-INSULIN received insulin therapy. For both studies, non-insulin glucose-lowering treatment included biguanides, sulfonylureas, dipeptidyl peptidase-4 inhibitors, glucagon-like peptide-1 receptor agonists, and sodium glucose contransporter-2 inhibitors.

### Efficacy

**Fig. 1:**
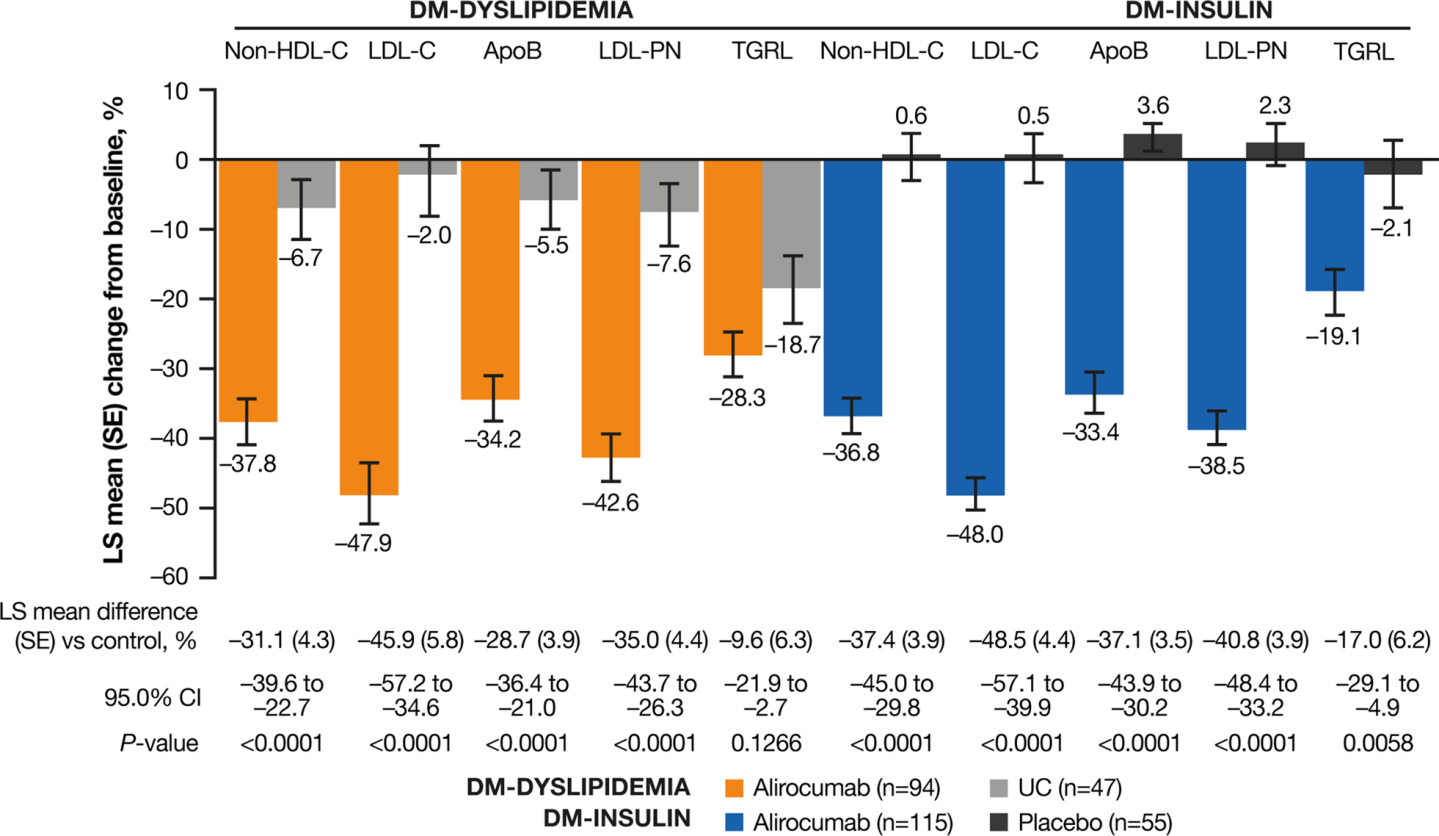
Percentage change from baseline to week 24 in non-HDL-C, LDL-C, ApoB, LDL-PN (ITT). *Apo* apolipoprotein, *HDL-C* high-density lipoprotein cholesterol, *ITT* intent-to-treat, *LDL-C* low-density lipoprotein cholesterol, *LDL-PN* low-density lipoprotein particle number, *LS* least-squares, *SE* standard error, *UC* usual care

**Fig. 2:**
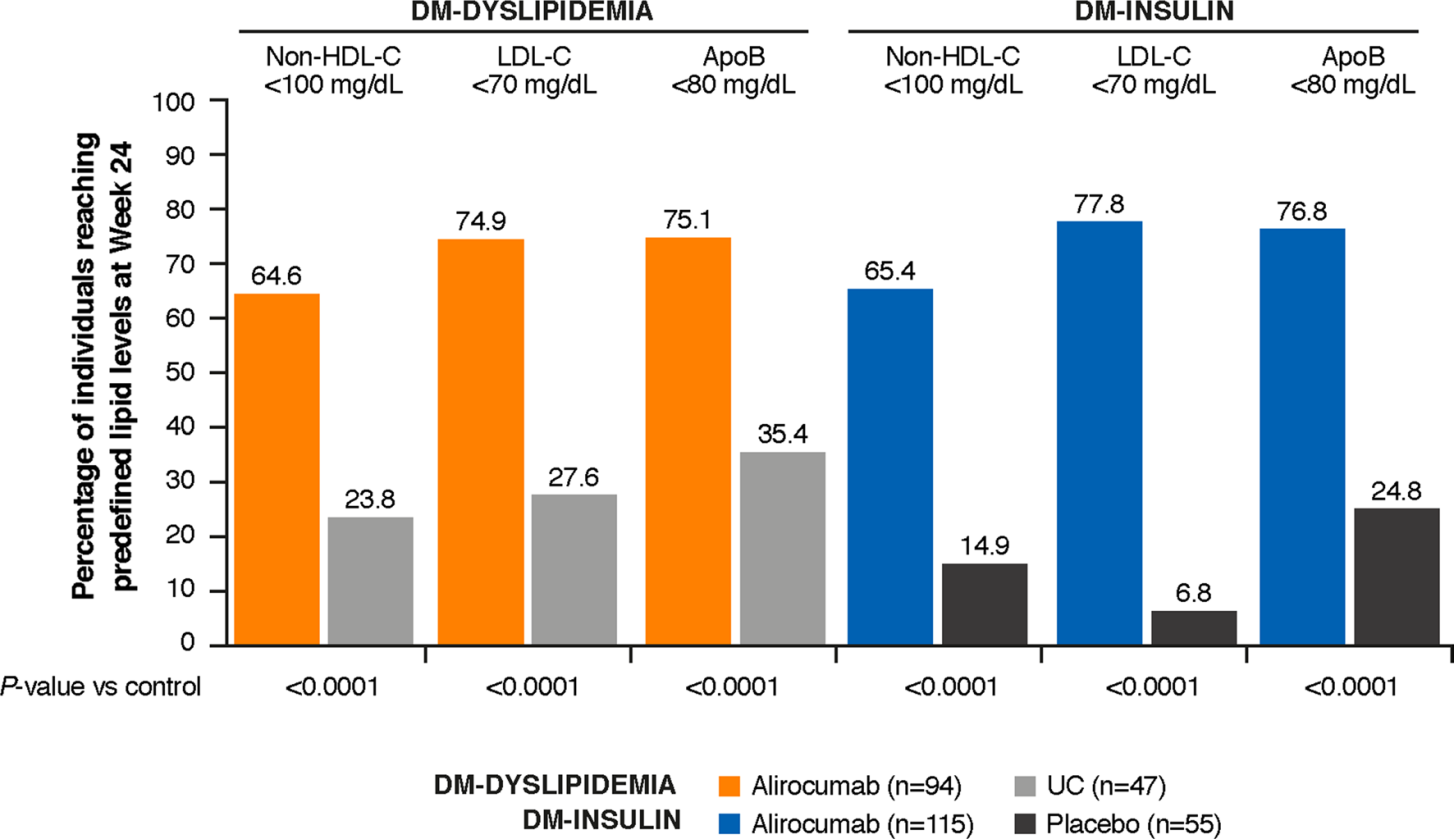
Percentage of individuals achieving non-HDL-C, LDL-C, and ApoB targets at week 24 (ITT). Non-HDL-C: 100 mg/dL = 2.59 mmol/L; LDL-C: 70 mg/dL = 1.81 mmol/L. *Apo* apolipoprotein, *HDL-C* high-density lipoprotein cholesterol, *ITT* intent-to-treat, *LDL-C* low-density lipoprotein cholesterol, *UC* usual care

Alirocumab significantly reduced non-HDL-C, LDL-C, ApoB, and LDL-PN from baseline to week 24 versus controls among individuals with T2DM and ASCVD in the ODYSSEY DM-DYSLIPIDEMIA and DM-INSULIN studies (Fig. 1). The percentage change from baseline to week 24 in LDL-PN was − 42.6% for alirocumab versus − 7.6% for UC in DM-DYSLIPIDEMIA and − 38.5% for alirocumab versus 2.3% for placebo (Fig. 1). The LS mean difference (standard error [SE]) versus control was − 35.0% (4.4) (95% confidence interval [CI] − 43.7 to − 26.3; *P *< 0.0001) for DM-DYSLIPIDEMIA and − 40.8% (3.9) (95% CI − 48.4 to − 33.2; *P *< 0.0001) for DM-INSULIN. Notably, the percentage change from baseline to week 24 in TGRL was − 28.3% (3.6) for alirocumab versus − 18.7% (5.1) for UC in DM-DYSLIPIDEMIA (adjusted mean difference [SE] − 9.6 [6.3] 95% confidence interval [CI] − 21.9 to 2.7, *P *= 0.13) and − 19.1% (3.5) for alirocumab versus − 2.1% (5.1) for placebo in DM-INSULIN (adjusted mean difference [SE] − 17.0 [6.2] 95% CI − 29.1 to − 4.9, *P *= 0.01). At week 24, a significantly greater proportion of individuals achieved non-HDL-C < 100 mg/dL (< 2.59 mmol/L), LDL-C < 70 mg/dL (< 1.81 mmol/L), and ApoB < 80 mg/dL versus control (all *P *< 0.0001; Fig. 2).

### Safety

In total, 66.7% (alirocumab) and 67.3% (control) of individuals reported TEAEs (Table 2). Treatment emergent serious adverse events (SAEs) were reported in 9.5% (alirocumab) versus 8.8% (UC) of individuals in DM-DYSLIPIDEMIA and 9.0% (alirocumab) versus 9.4% (placebo) of individuals in DM-INSULIN. The adverse event pattern was similar in both pooled alirocumab and control groups. Results for adverse events for the two treatment arms in each study may be seen in Table 3.

**Table 2:** Safety summary (pool of DM-INSULIN and DM-DYSLIPIDEMIA; safety population)

n (%)	Alirocumab (n = 213)	Control (n = 104)
TEAEs	142 (66.7)	70 (67.3)
Treatment-emergent SAEs	28 (13.1)	10 (9.6)
TEAEs leading to death	1 (0.5)	1 (1.0)
TEAEs occurring in ≥ 2% of individuals by preferred term		
Urinary tract infection	8 (3.8)	6 (5.8)
Diarrhea	8 (3.8)	6 (5.8)
Hypertension	8 (3.8)	4 (3.8)
Influenza	7 (3.3)	4 (3.8)
Headache	7 (3.3)	1 (1.0)
Musculoskeletal pain	7 (3.3)	3 (2.9)
Nasopharyngitis	6 (2.8)	5 (4.8)
Back pain	6 (2.8)	2 (1.9)
Dizziness	6 (2.8)	3 (2.9)
Fatigue	5 (2.3)	3 (2.9)
Cataract	5 (2.3)	1 (1.0)
Myalgia	5 (2.3)	1 (1.0)
Nausea	4 (1.9)	3 (2.9)
Pain in extremity	4 (1.9)	3 (2.9)
Arthralgia	3 (1.4)	3 (2.9)
Bronchitis	3 (1.4)	3 (2.9)
Hypotension	2 (0.9)	3 (2.9)
Cough	1 (0.5)	3 (2.9)
Hyperglycemia	0 (0.0)	3 (2.9)

*SAE* serious adverse event, *TEAE* treatment-emergent adverse event

**Table 3:** Safety summary (randomized population)

	DM-DYSLIPIDEMIA		DM-INSULIN	
	Alirocumab (n = 95)	UC (n = 47)	Alirocumab (n = 118)	Placebo (n = 57)
TEAEs occurring in ≥ 2% of individuals by preferred term, n (%)				
Urinary tract infection	5 (5.3)	2 (4.3)	3 (2.5)	4 (7.0)
Diarrhea	5 (5.3)	3 (6.4)	3 (2.5)	3 (5.3)
Hypertension	3 (3.2)	1 (2.1)	5 (4.2)	3 (5.3)
Influenza	3 (3.2)	3 (6.4)	4 (3.4)	1 (1.8)
Musculoskeletal pain	4 (4.2)	1 (2.1)	3 (2.5)	2 (3.5)
Back pain	2 (2.1)	2 (4.3)	4 (3.4)	0
Dizziness	3 (3.2)	1 (2.1)	3 (2.5)	2 (3.5)
Fatigue	2 (2.1)	2 (4.3)	3 (2.5)	1 (1.8)
Cataract	1 (1.1)	1 (2.1)	4 (3.4)	0
Myalgia	2 (2.1)	1 (2.1)	3 (2.5)	0
Nausea	2 (2.1)	1 (2.1)	2 (1.7)	2 (3.5)
Pain in extremity	2 (2.1)	2 (4.3)	2 (1.7)	1 (1.8)
Bronchitis	0	2 (4.3)	3 (2.5)	1 (1.8)

Mean (SD) levels of HbA1c were similar in each treatment group at baseline (alirocumab: 7.3 [0.9]%; control: 7.3 [0.9]%) and week 24 (alirocumab: 7.6 [1.2]%; control: 7.5 [1.2]%; safety analysis). Fasting plasma glucose levels were also similar regardless of treatment allocation at baseline (alirocumab: 154.2 [47.9] mg/dL, 8.6 [2.7] mmol/L; control: 149.5 [43.7] mg/dL, 8.3 [2.4] mmol/L) and at week 24 (alirocumab: 164.7 [54.9] mg/dL, 9.1 [3.0] mmol/L; control: 159.4 [48.4] mg/dL, 8.9 [2.7] mmol/L; safety analysis).

## Discussion

### Efficacy and safety

The participants included in this analysis from the DM-DYSLIPIDEMIA and DM INSULIN trials had both prior ASCVD and T2DM, and represent a population with a very high risk of CV events for which a target LDL-C < 70 mg/dL and non-HDL-C < 100 mg/dL were generally recommended at the time of study implementation [10, 11]. In our study population, statins at maximally tolerated doses were largely insufficient to achieve guideline-recommended lipid goals and thus it represents a group with high residual risk and an unmet therapeutic need. In the present analysis, significant reductions in LDL-C, non-HDL-C, and Apo B were observed with alirocumab versus controls, which is consistent with the results in the overall trial populations from the primary DM-DYSLIPIDEMIA and DM-INSULIN studies. Alirocumab treatment resulted in significant LDL-C reductions from baseline to week 24 compared with either UC or placebo (− 43.0% and − 49.0%, respectively; *P *< 0.0001) on background maximally tolerated statin therapy [4, 8]. Furthermore, alirocumab also significantly reduced non-HDL-C and ApoB at week 24 versus control, which better predict total atherogenic burden in this population, and results were consistent with the results of the primary studies [4, 8]. Finally, levels of low-density lipoprotein particle (LDL-P), which may more closely align with CV risk than LDL-C in diabetes, were also significantly reduced with alirocumab therapy.

This post hoc analysis confirmed similar adverse event patterns in alirocumab-treated individuals and controls already reported in other studies. In both studies, alirocumab was generally well tolerated, with comparable rates of TEAEs between alirocumab and UC or placebo. The overall incidence of injection-site reactions for both primary studies was low, with no greater incidence seen in alirocumab-treated individuals [4, 8]. However, in the overall alirocumab trial population, including both individuals with and without diabetes, injection-site reactions were found at a higher frequency with alirocumab compared with controls [8, 12]. In a real-world analysis of three datasets from a hospital registry (n = 164), and two pharmacovigilance databases, Lareb (n = 149) and VigiLyze (n = 15,554), PCSK9 inhibitors were found to be well tolerated with an overall safety profile comparable to currently available randomized clinical trials [13]. The most common adverse events included influenza-like illness, nasopharyngitis, myalgia, and injection-site reactions, all of which resolved over time. Furthermore, while the benefit of PCSK9 inhibitors is primarily ascribed to their LDL-C reducing activity, data suggest that they may also influence platelet function and blood coagulation [14].

### CV events and outcomes

Long-term CV outcome studies have shown that PCSK9 inhibitors reduce CV event rates similarly in individuals with and without T2DM and prior CV disease or recent acute coronary syndrome [15, 16]. However, absolute risk is higher among those with diabetes and established ASCVD and therefore these patients are expected to derive greater absolute benefits from further LLT. In the FOURIER trial, patients with diabetes and a baseline LDL-C level of around 90 mg/dL had a 5-point major adverse CV event rate of 17.1%, which decreased to 14.4% by reducing LDL-C to around 30 mg/dL [17]. The latter event rate is only marginally higher than the 13.0% event rate among patients without diabetes, stable ASCVD, and LDL-C of around 90 mg/dL, highlighting the important role more intensive LDL-C lowering can have in mitigating the excess CV risk in high-risk states. In addition, a reduction in LDL-C levels to target concentrations of 25.1 mg/dL (0.65 mmol/L)–50.3 mg/dL (1.3 mmol/L) has been proposed to reduce CV events in patients with a recent ACS and diabetes [15]. In an analysis of the ODYSSEY OUTCOMES trial, the effect of alirocumab on CV outcomes was assessed by baseline glycemic status (27.7% were patients with normoglycemia, 28.8% with diabetes, and 43.6% with pre-diabetes). Results demonstrated that after a recent ACS, treatment with alirocumab targeting the above-mentioned LDL-C concentrations produces about twice the absolute reduction in CV events among patients with diabetes as in those without diabetes [15]. Furthermore, it has recently been reported that alirocumab on top of intensive statin therapy potentially reduces death after ACS, particularly in treatments for ≥ 3 years, if baseline LDL-C is ≥ 100 mg/dL, or if achieved LDL-C is low [18].

Current treatment guidelines recommend that LDL-C targets are set according to an individual’s CV risk. Individuals with T2DM and prior ASCVD are classified as being at extreme risk by the American Association of Clinical Endocrinologists (AACE), who recommend LDL-C targets of < 55 mg/dL, non-HDL-C targets of < 80 mg/dL, and ApoB targets of < 70 mg/dL for this patient population [19]. In addition, the AACE recommends the consideration of PCSK9 inhibitors in individuals with clinical CV disease who are unable to reach LDL-C or non-HDL-C goals with maximally tolerated statin therapy [19]. The published data from the FOURIER and ODYSSEY OUTCOMES trials further support this assertion; however, these more aggressive lipid targets have not been adopted by all guidelines to date. It is also important to note the influence that certain diabetic medications may have on PCSK9 expression. Liraglutide, a once-daily glucagon-like peptide-1 (GLP-1) agonist, was found to suppress PCSK9 expression via a hepatocyte nuclear factor 1 alpha (HNF1α)-dependent mechanism in the human hepatoma cell line, HepG2 [20]. In this analysis, between 9 and 17% of patients received a GLP-1 agonist; however, it would not be appropriate to speculate the effects this may exert on the results.

It is noteworthy that in DM-DYSLIPIDEMIA, alirocumab was superior to moderate-dose fish oils and fenofibrate in improving the atherogenic lipid profile [4]. Furthermore, results from the ACCORD-LIPID study demonstrated that combination therapy with fenofibrate and simvastatin did not significantly reduce CV event rates compared with statin monotherapy in patients with T2DM [21]. Ongoing trials, including the CVOT PROMINENT (NCT03071692) study are further investigating the use of fibrates in this patient population. This is clinically relevant as fish oil and fenofibrate are considered by some as therapeutic options after statins in patients with mixed dyslipidemia. With that said, high dose eicosapentaenoic acid in the REDUCE-IT trial did reduce CV events despite achieving only modest reductions in triglycerides and ApoB [22]. The reasons for this are unclear but could include beneficial pleiotropic effects beyond the change in atherogenic particle number.

### Analysis of DM-DYSLIPIDEMIA and DM-INSULIN

DM-DYSLIPIDEMIA and DM-INSULIN were separate trials with different study designs (one versus UC and the other versus placebo); however, both studies were conducted exclusively in patients with diabetes. While patients in the DM-DYSLIPIDEMIA trial were enrolled because of elevated non-HDL-C, those in DM-INSULIN were enrolled only based on elevated LDL-C. In DM-DYSLIPIDEMIA, alirocumab significantly reduced non-HDL-C (the primary endpoint) and LDL-C versus UC (*P *< 0.0001) [4]. In DM-INSULIN, alirocumab treatment resulted in significant LDL-C reductions compared with placebo (*P *< 0.0001) [8]. As the study designs were different, we presented our analyses separately for each trial. However, it is worth noting that patients in the DM-INSULIN trial had similar ApoB and LDL-P levels to those patients in the DM-DYSLIPIDEMIA trial. Furthermore, insulin therapy is often the end stage of glucose-lowering medication intensification and the observation that patients on insulin had high levels of atherogenic lipoproteins only serves to reinforce the high CV risk of this patient population.

One major rationale for evaluating the subgroups with ASCVD in DM-DYSLIPIDEMIA and DM-INSULIN is because both studies investigated patients with increased levels of TGRL (non-HDL-C minus LDL-C). The average baseline level of TGRL was approximately 34 mg/dL and 48 mg/dL in DM-INSULIN and DM-DYSLIPIDEMIA, respectively [4, 8]. These studies therefore represent a different diabetic patient population than those included in the FOURIER and ODYSSEY OUTCOMES trials, with broader eligibility criteria regarding the type of CV disease. In both of these clinical outcomes trials, non-HDL-C levels were ≤ 125 mg/dL and TGRL levels were < 32 mg/dL [7, 16].

## Conclusion

In summary, among very high risk individuals with T2DM and ASCVD who had elevated non-HDL-C (DM-DYSLIPIDEMIA) or LDL-C (DM-INSULIN) levels despite maximally tolerated statins, alirocumab significantly reduced atherogenic cholesterol and improved achievement of LDL-C, non-HDL-C, and ApoB goals versus controls.

## Abbreviations

AACE: American Association of Clinical Endocrinologists
ACS: acute coronary syndrome
ADA: American Diabetes Association
ApoB: apolipoprotein B
ASCVD: atherosclerotic cardiovascular disease
CHD: coronary heart disease
CV: cardiovascular
EAS: European Atherosclerosis Society
ESC: European Cardiovascular Society
HDL-C: high-density lipoprotein cholesterol
LDL-C: low-density lipoprotein cholesterol
LDL-PN: low-density lipoprotein particle number
LLT: lipid-lowering therapy
MI: myocardial infarction
PAD: peripheral artery disease
PCSK9: proprotein convertase subtilisin/kexin type 9
Q2W: every 2 weeks
T1DM: type 1 diabetes mellitus
T2DM: type 2 diabetes mellitus
TEAE: treatment-emergent adverse event
UC: usual care

## Availability of data and materials

Qualified researchers may request access to patient level data and related study documents including the clinical study report, study protocol with any amendments, blank case report form, statistical analysis plan, and dataset specifications. Patient level data will be anonymized and study documents will be redacted to protect the privacy of trial participants. Further details on Sanofi’s data sharing criteria, eligible studies, and process for requesting access can be found at: https://www.clinicalstudydatarequest.com.
